# Supplementary material for: Role of Interfacial Morphology in Cu2O/TiO2 and Band Bending: Insights from Density Functional Theory
Source: ACS Appl Mater Interfaces. 2024 Jun 26;16(27):35781–92. doi: 10.1021/acsami.4c06081 (PMC11247431; doi:10.1021/acsami.4c06081)
Supplement: Supplementary file 1 — am4c06081_si_001.pdf [file am4c06081_si_001.pdf]

Supporting information file for:

The Role of Interfacial Morphology in Cu_2O/TiO_2 and Band Bending: Insights from Density Functional Theory

Mona Asadinamin,[1] Aleksandar Živkovic[2,3], Nora H. De Leeuw[2,4], and Steven P. Lewis[1]

[1] *Department of Physics and Astronomy, University of Georgia, Athens, Georgia 30602, US*

[2] *Department of Earth Sciences, Utrecht University, Princetonlaan 8a, 3548CB Utrecht, The Netherlands*

[3] *Institute of Inorganic Chemistry, Kiel University, 24118 Kiel, Germany*

[4] *School of Chemistry, University of Leeds, Leeds LS2 9JT, UK*

Corresponding author: a.zivkovic@uu.nl, splewis@uga.edu

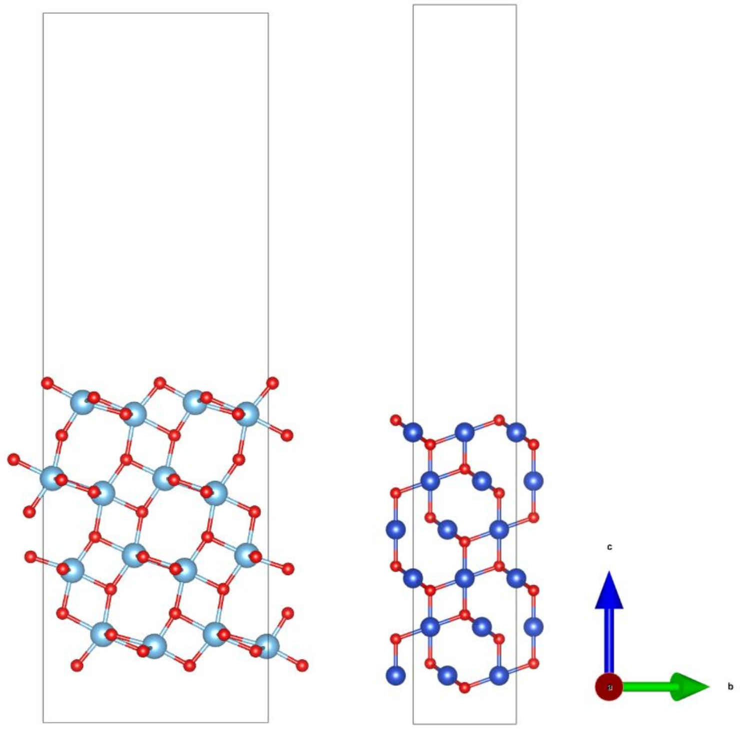

Figure S1. Side view of the slab morphologies of the 4-bilayer TiO_2 (101) surface (left) and 6-trilayer Cu_2O (111) surface (right). Ti: light blue, oxygen: red, and cu: dark blue. The coordinate system is shown by the blue and green arrows where c indicates the non-periodic direction.

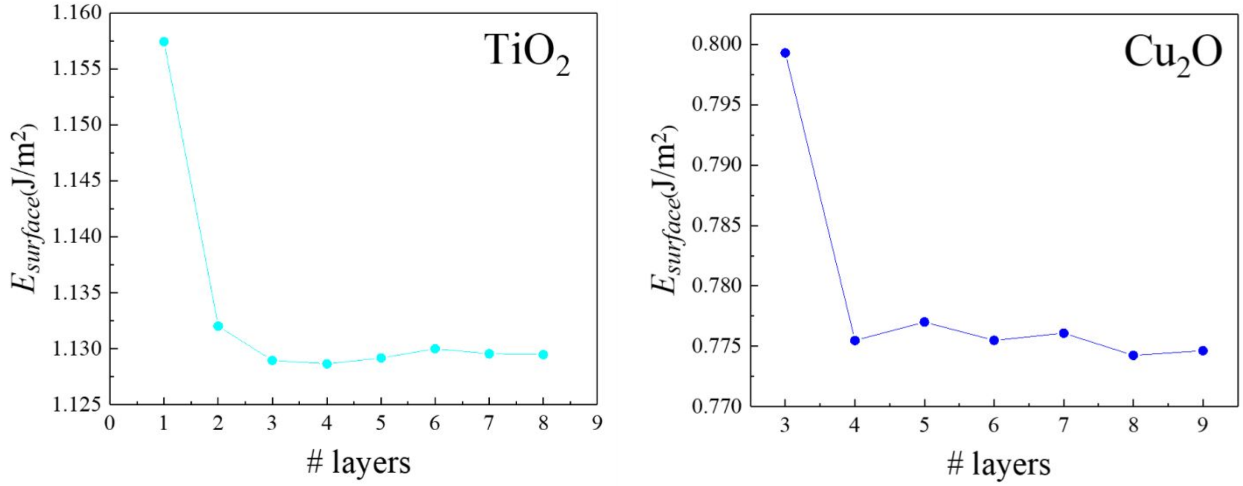

Figure S2. Surface energies of the relaxed TiO_2 (left) and Cu_2O (right) slabs which quickly converged to a value of approximately 1.22 J/m2 for a 4-bilayer TiO_2 and 0.77 J/m2 for a 4-trilayer Cu_2O.

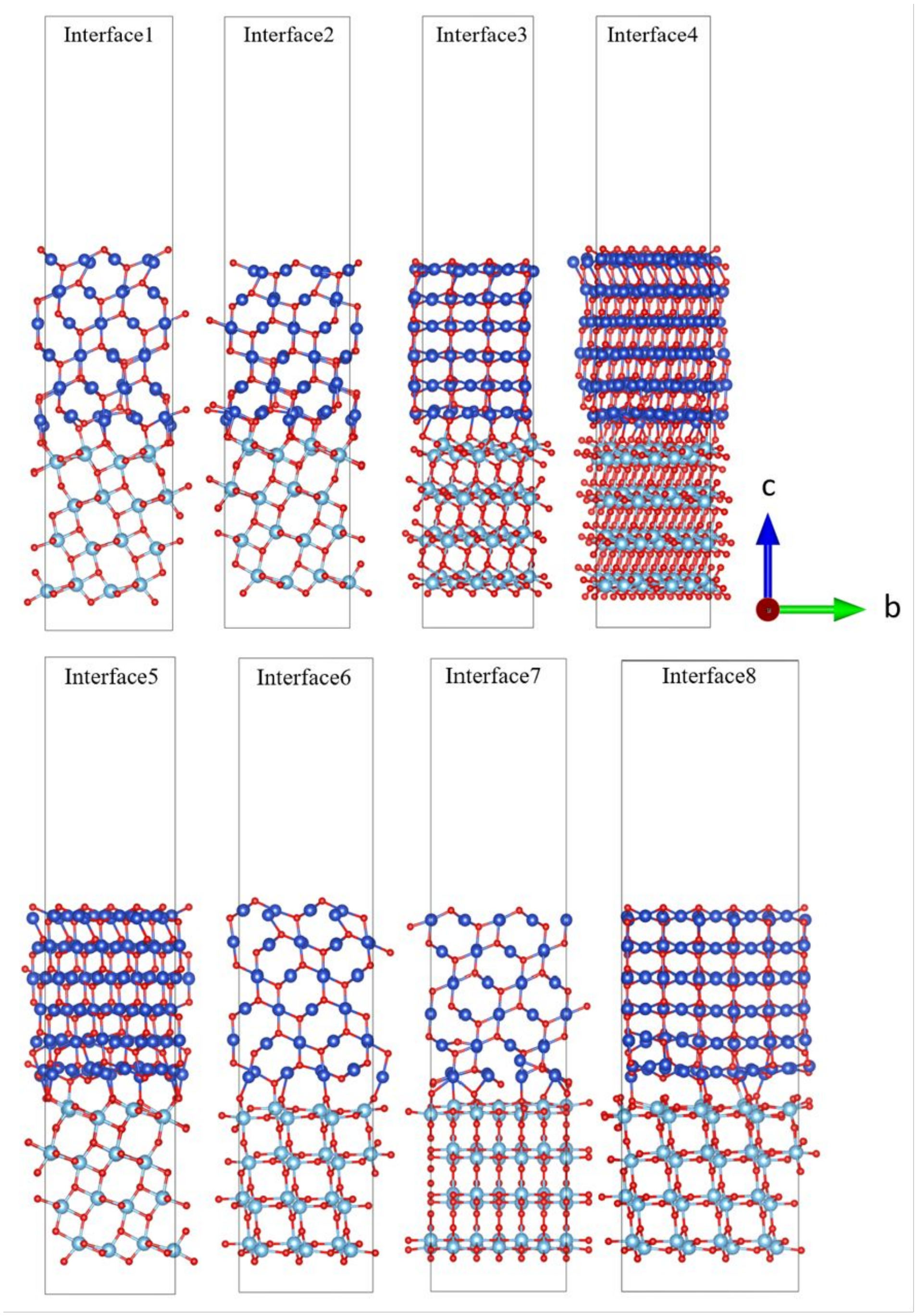

Figure S3. Side view unit cell of the relaxed structure of interfaces 1-8. The green and blue arrows show the coordinate systems.

Table S1. Computed values of electronic band gap, ionization potential (IP), and electron affinity (EA) for Cu2O and TiO2.

Compound	XC	Kohn-Sham gap (eV)	IP (eV)	EA (eV)	Exp. (eV)
Cu_2O	PBE	0.46	4.64	4.18	5.0 eV to 4.0 eV (IP[1]) 3.20 eV (EA[2])
	HSE06	1.94	5.81	3.87	
TiO_2	PBE	1.94	7.00	5.03	7.96 (IP[3]) 5.1 to 5.3 eV (EA[4])
	HSE06	3.34	8.84	5.30	

Table S2. Computed valence band and conduction band offsets at the Cu_2O / TiO_2 interface using two different approaches, the independent compounds alignment and the alignment based on an explicitly modelled interfacial structure.

System	XC	Valence band offset (independent compound alignment) (eV)	Conduction band offset (independent compound alignment) (eV)	Valence band offset (explicit interface) (eV)	Conduction band offset (explicit interface) (eV)
$Cu_2O(111)$ / $TiO_2(101)$	PBE	2.35	0.84	1.93	0.42
	HSE06	2.83	1.43	1.92[1]	0.52

[1] The potential offset at the interface was taken from PBE calculations, assuming transferability of values as outlined in the work of (5) Conesa, J. C. Modeling with Hybrid Density Functional Theory the Electronic Band Alignment at the Zinc Oxide–Anatase Interface. *The Journal of Physical Chemistry C* **2012**, *116* (35), 18884-18890. DOI: 10.1021/jp306160c..

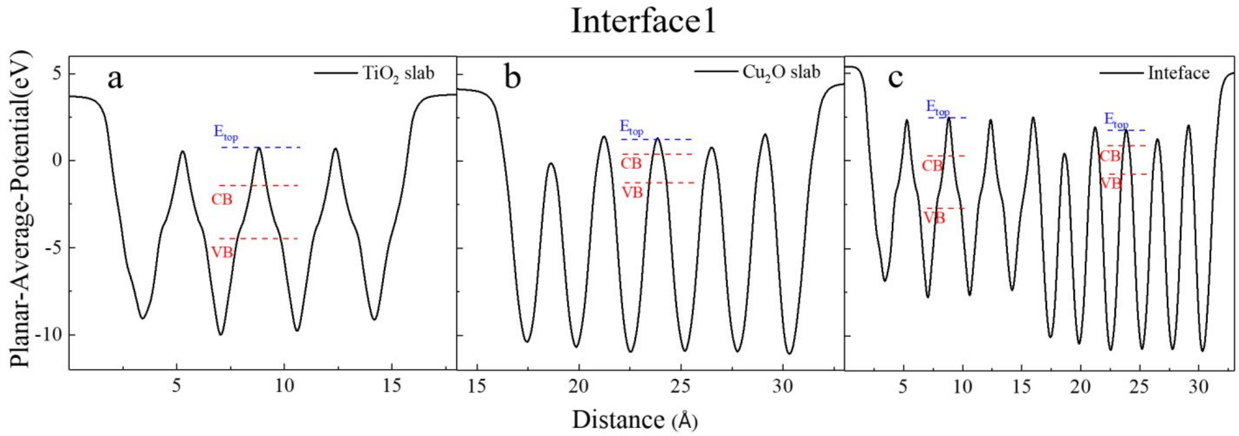

Figure S4. planar-averaged electrostatic potentials in the x-y plane of (a,b) the isolated slabs with the interface geometries, and (c) of interface 1 as a function of the distance along the z-direction, normal to the interface.

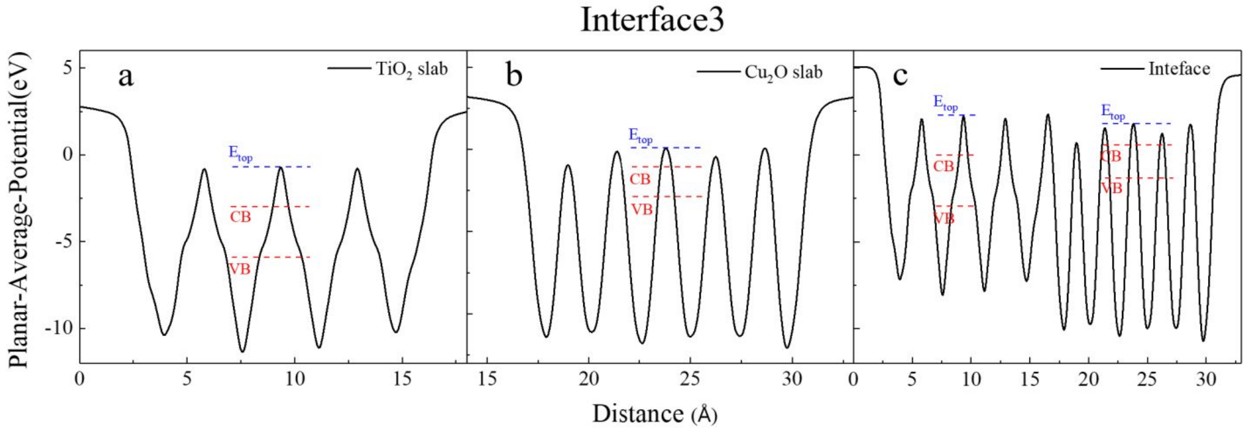

Figure S5. planar-averaged electrostatic potentials in the x-y plane of (a,b) the isolated slabs with the interface geometries, and (c) of interface 3 as a function of the distance along the z-direction, normal to the interface.

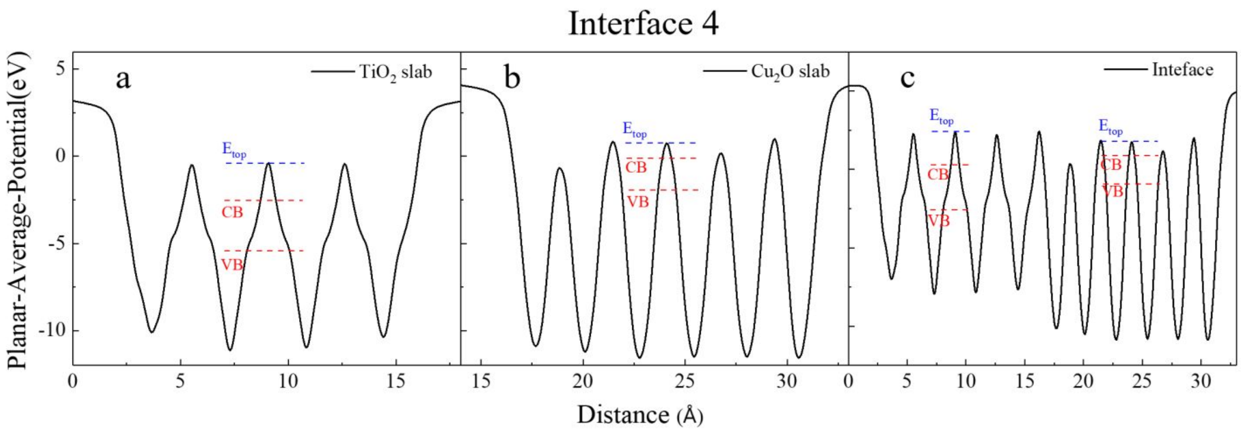

Figure S6. planar-averaged electrostatic potentials in the x-y plane of (a,b) the isolated slabs with the interface geometries, and (c) of interface 4 as a function of the distance along the z-direction, normal to the interface.

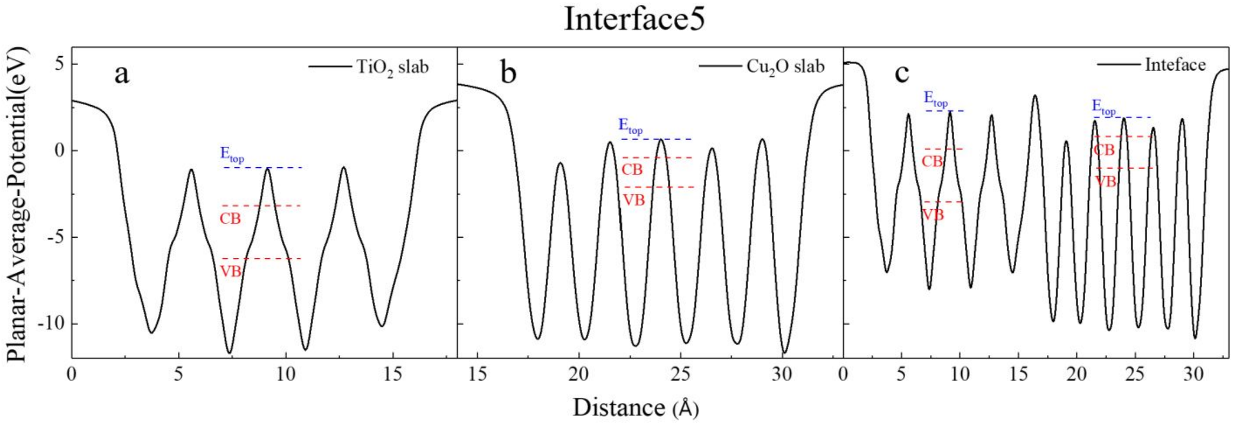

Figure S7. planar-averaged electrostatic potentials in the x-y plane of (a,b) the isolated slabs with the interface geometries, and (c) of interface 5 as a function of the distance along the z-direction, normal to the interface.

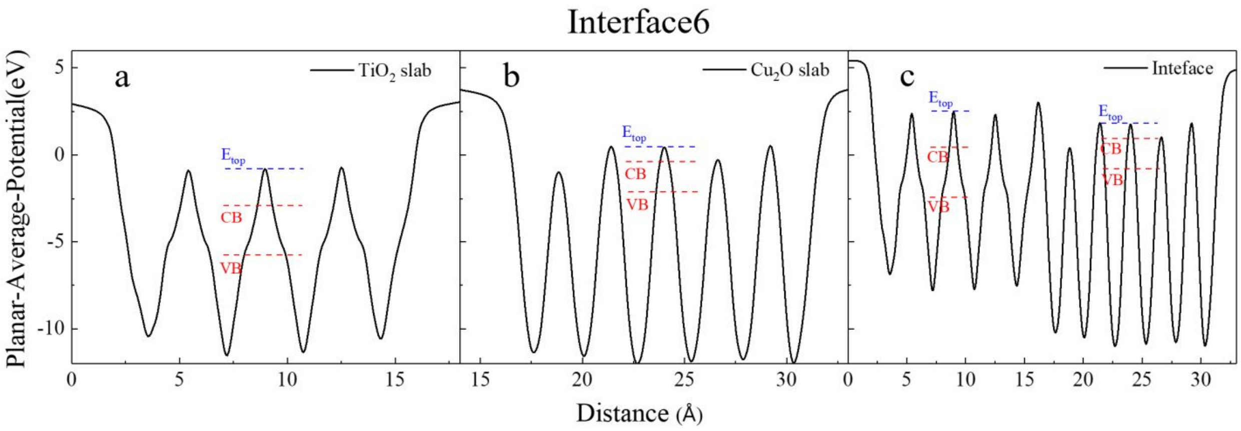

Figure S8. planar-averaged electrostatic potentials in the x-y plane of (a,b) the isolated slabs with the interface geometries, and (c) of interface 6 as a function of the distance along the z-direction, normal to the interface.

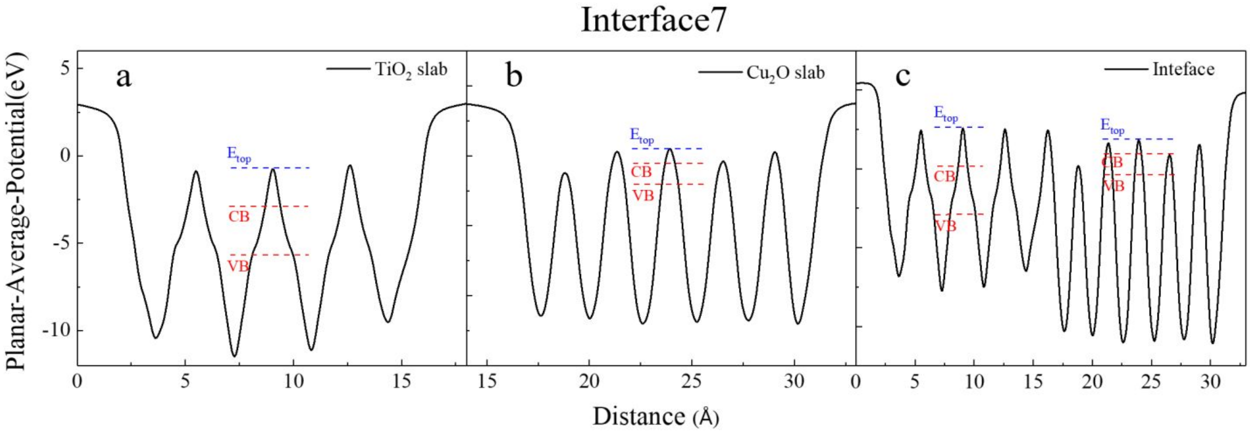

Figure S9. planar-averaged electrostatic potentials in the x-y plane of (a,b) the isolated slabs with the interface geometries, and (c) of interface 7 as a function of the distance along the z-direction, normal to the interface.

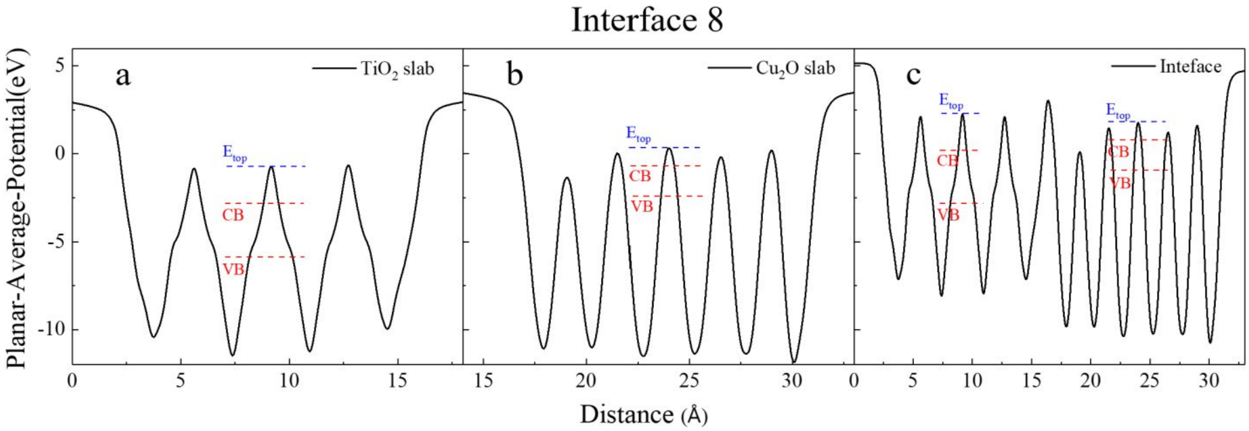

Figure S10. planar-averaged electrostatic potentials in the x-y plane of (a,b) the isolated slabs with the interface geometries, and (c) of interface 8 as a function of the distance along the z-direction, normal to the interface.

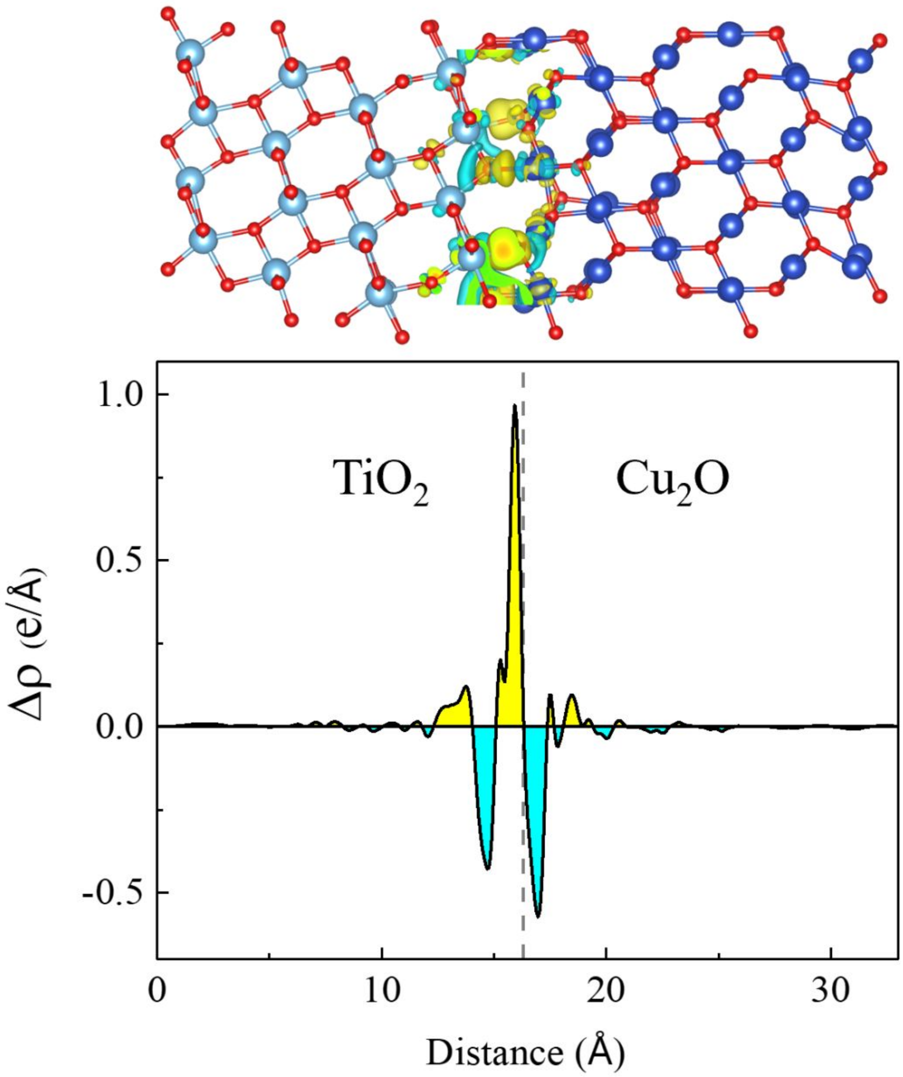

Figure S11. Charge density difference of interface 1. Yellow: charge accumulation. Cyan: charge depletion. The dashed line represents the midpoint layer of the interface.

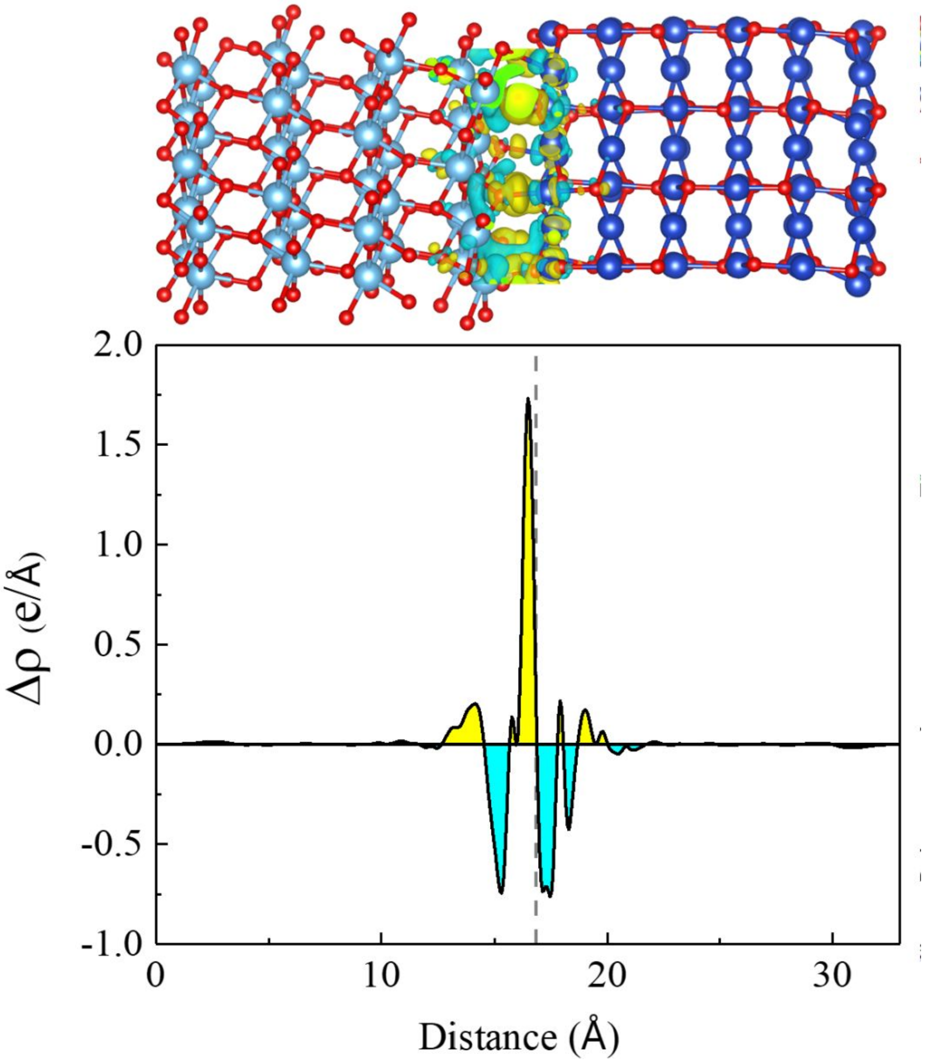

Figure S12. Charge density difference of interface 3. Yellow: charge accumulation. Cyan: charge depletion. The dashed line represents the midpoint layer of the interface.

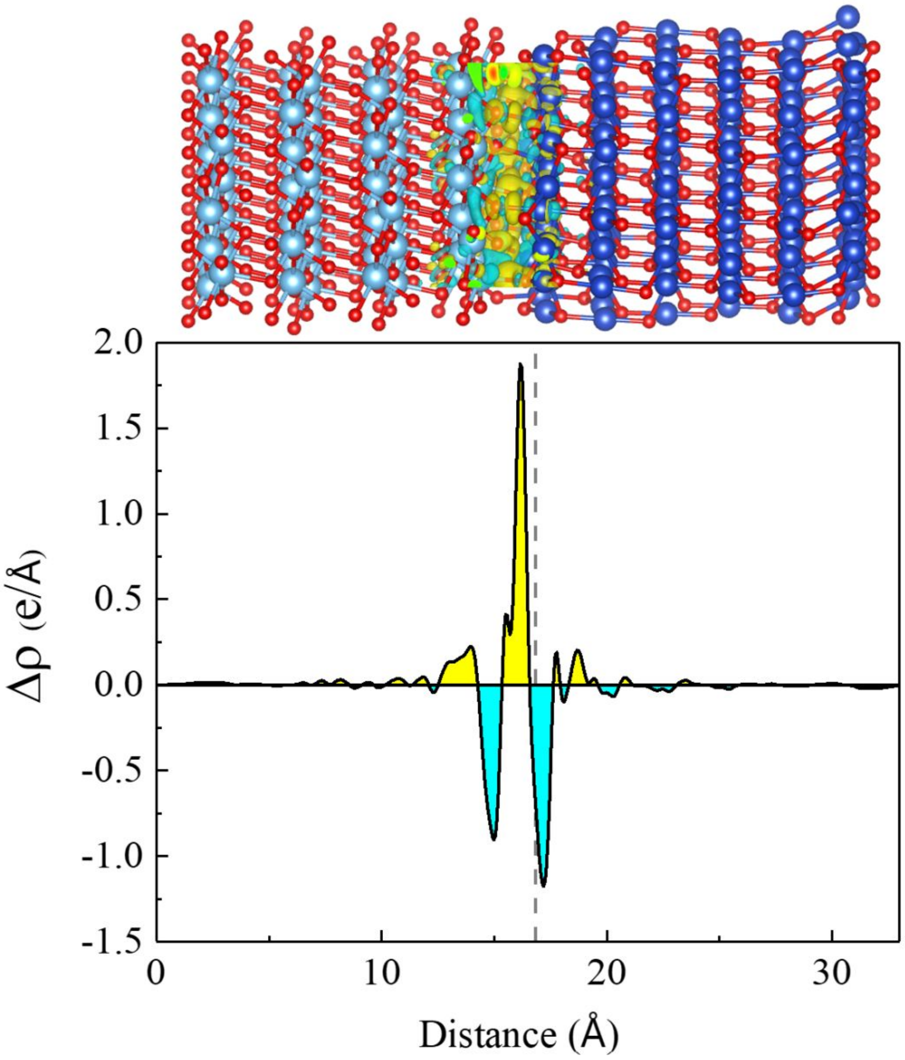

Figure S13. Charge density difference of interface 4. Yellow: charge accumulation. Cyan: charge depletion. The dashed line represents the midpoint layer of the interface.

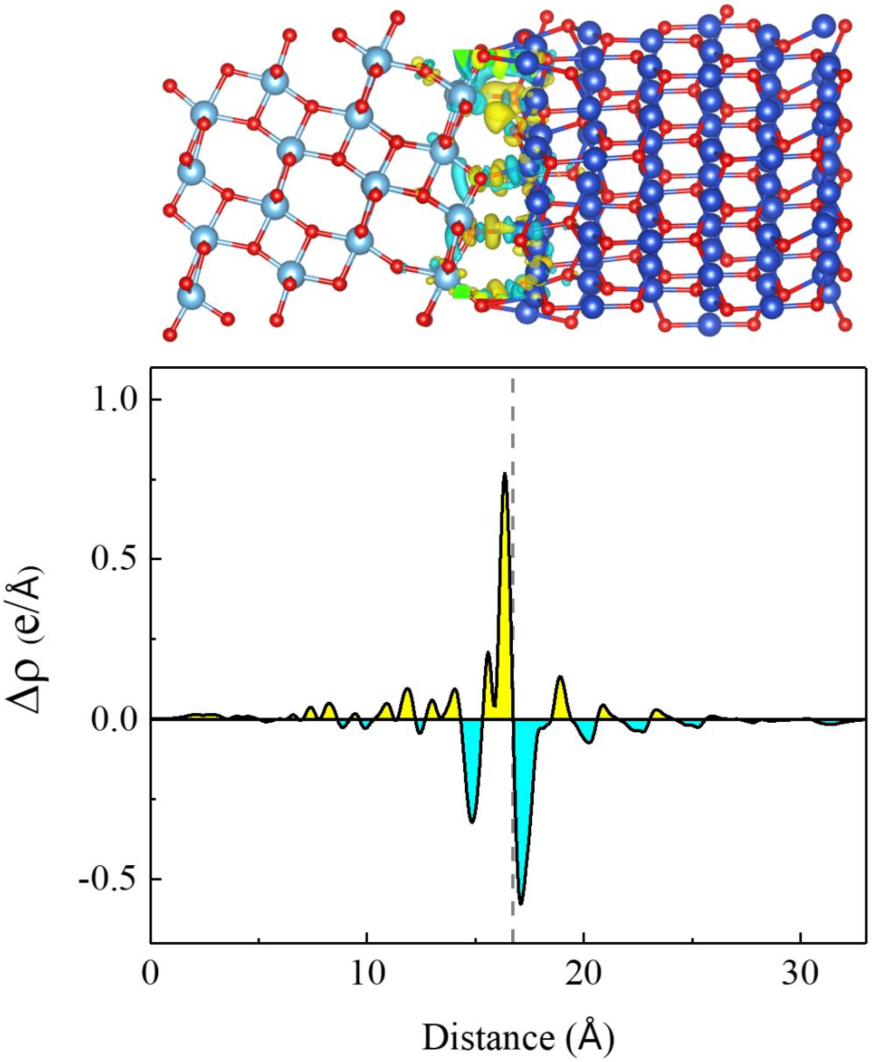

Figure S14. Charge density difference of interface 5. Yellow: charge accumulation. Cyan: charge depletion. The dashed line represents the midpoint layer of the interface.

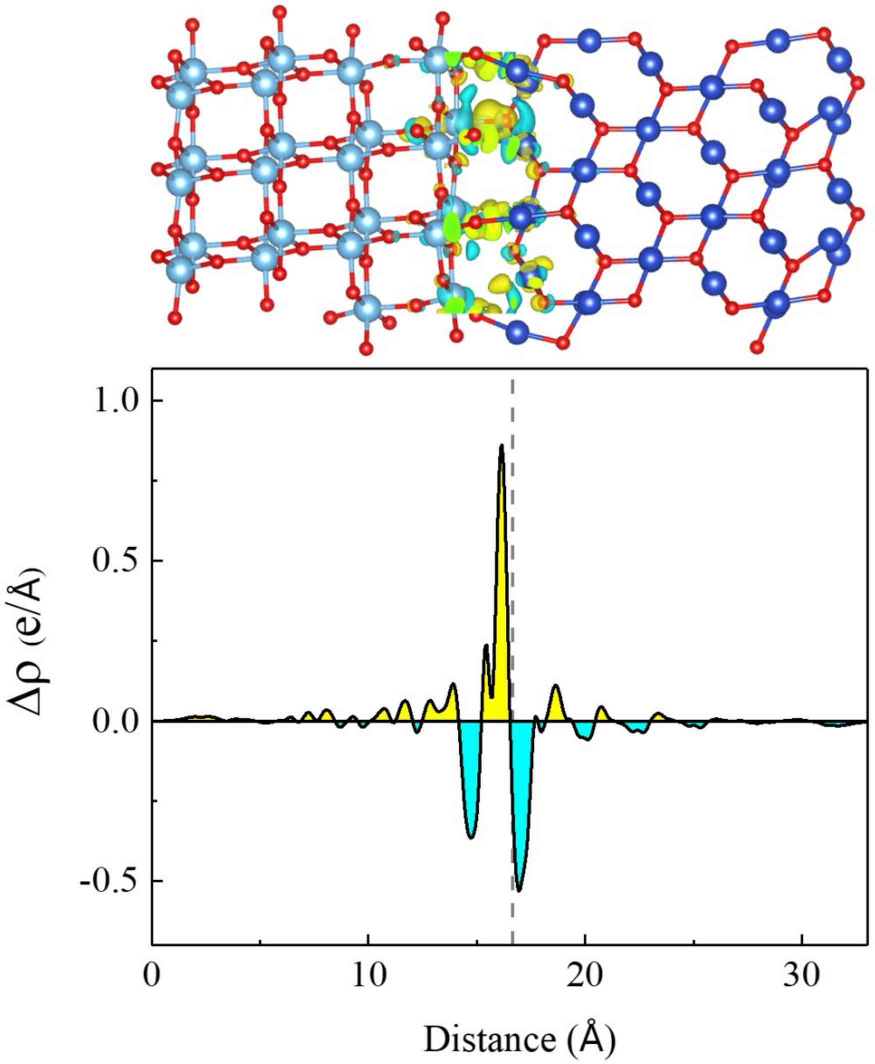

Figure S15. Charge density difference of interface 6. Yellow: charge accumulation. Cyan: charge depletion. The dashed line represents the midpoint layer of the interface.

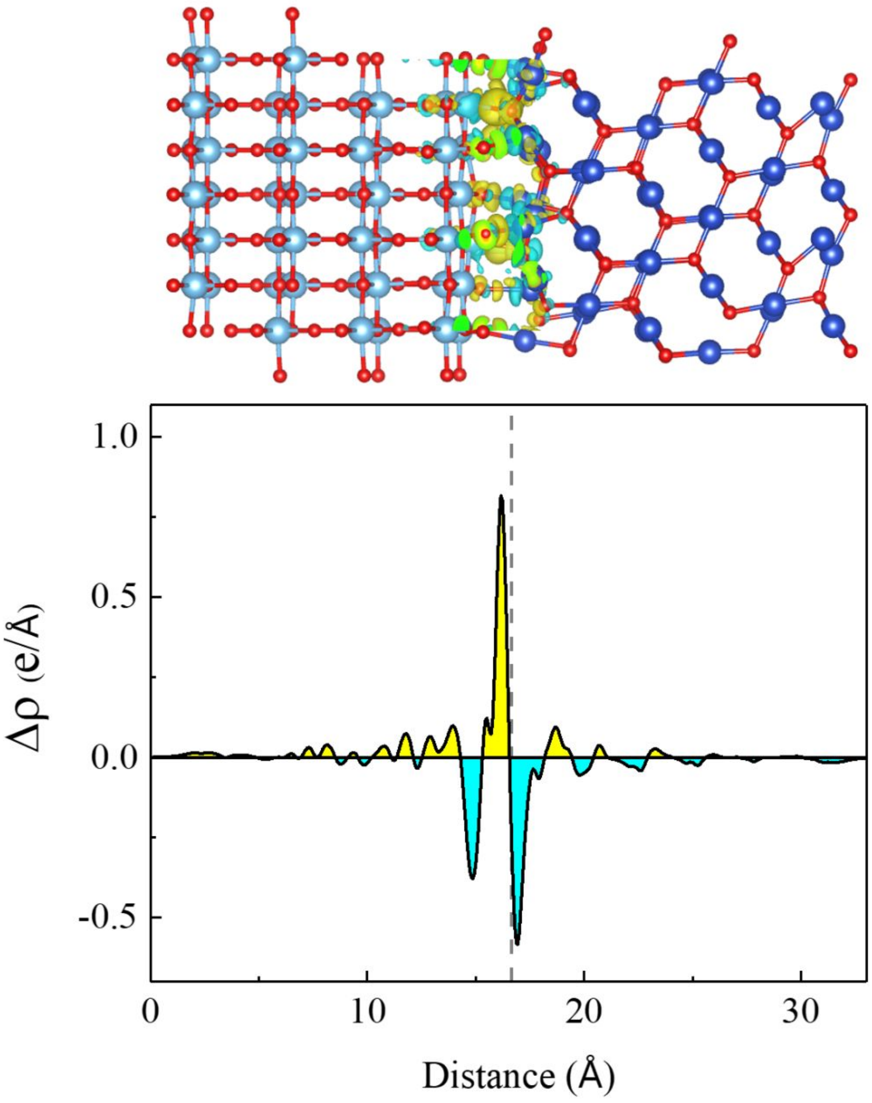

Figure S16. Charge density difference of interface 7. Yellow: charge accumulation. Cyan: charge depletion. The dashed line represents the midpoint layer of the interface.

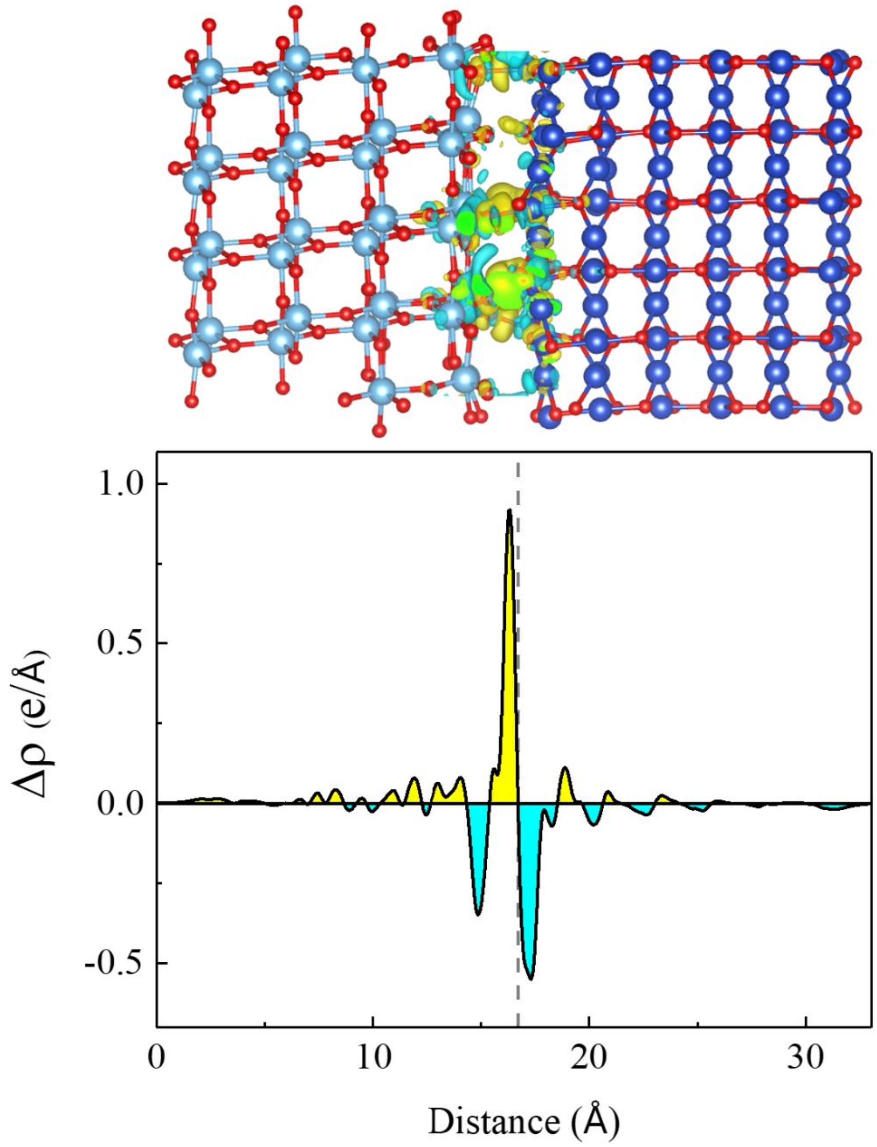

Figure S17. Charge density difference of interface 8. Yellow: charge accumulation. Cyan: charge depletion. The dashed line represents the midpoint layer of the interface.

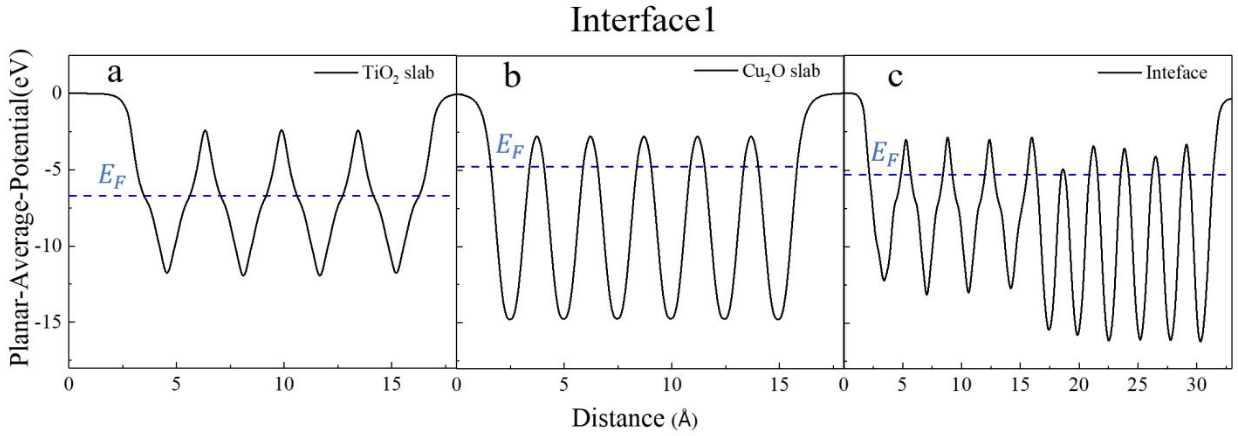

Figure S18. Fermi energy assessment of interface 1 (a,b) before and (c) after contact

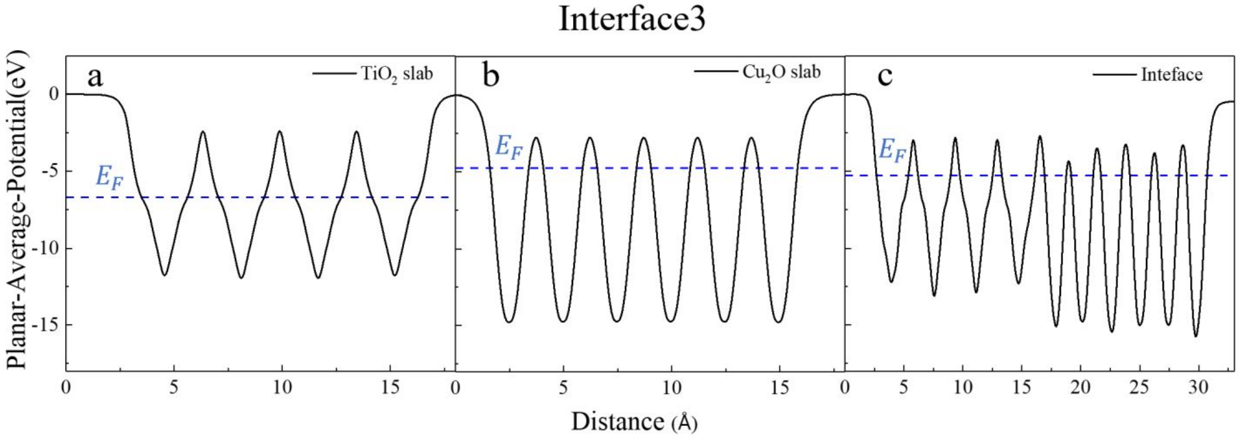

Figure S19. Fermi energy assessment of interface 3 (a,b) before and (c) after contact

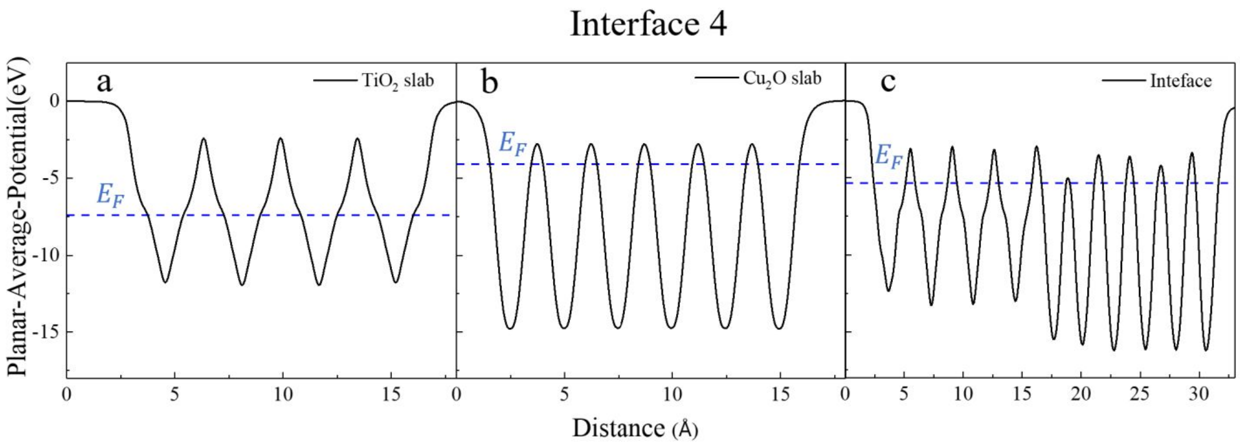

Figure S20. Fermi energy assessment of interface 4 (a,b) before and (c) after contact

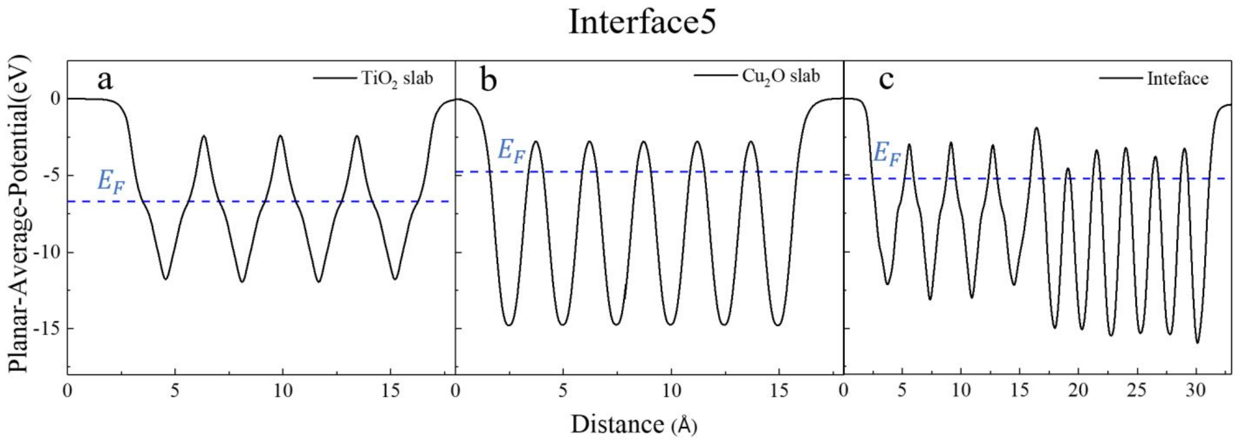

Figure S21. Fermi energy assessment of interface 5 (a,b) before and (c) after contact

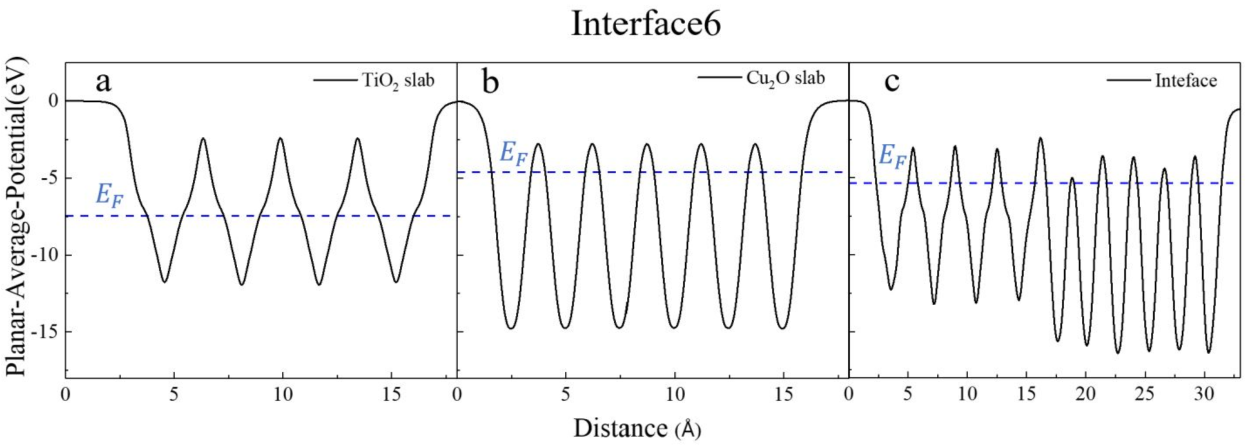

Figure S22. Fermi energy assessment of interface 6 (a,b) before and (c) after contact

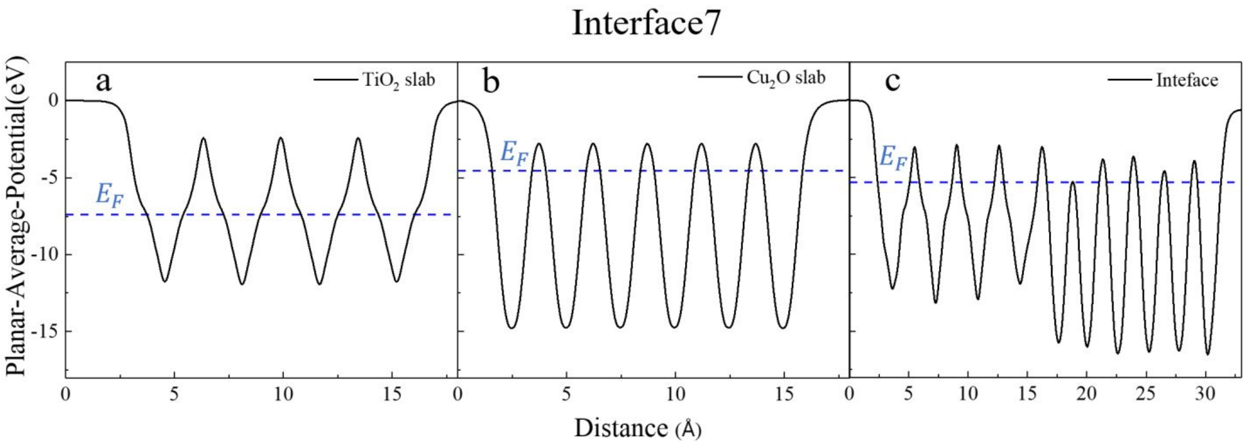

Figure S23. Fermi energy assessment of interface 7 (a,b) before and (c) after contact

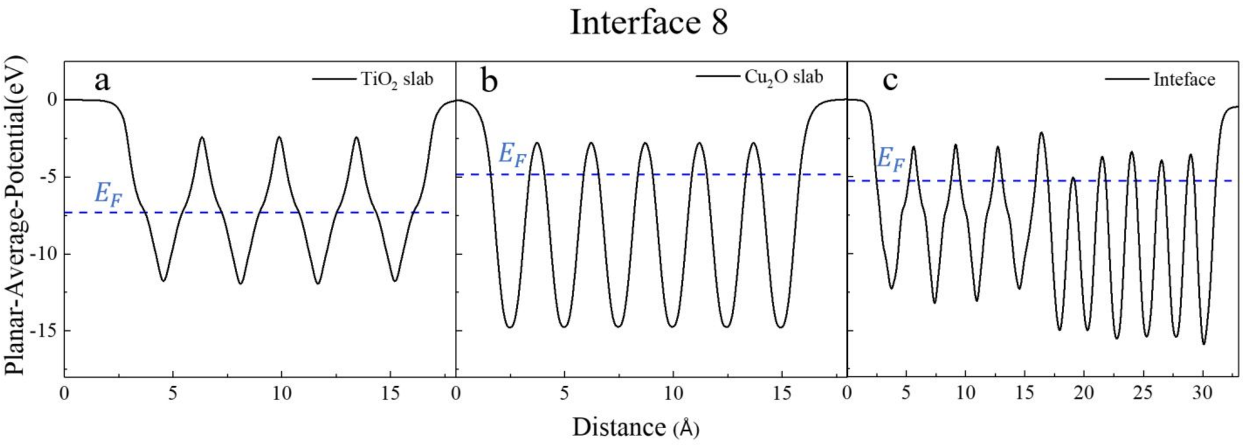

Figure S24. Fermi energy assessment of interface 8 (a,b) before and (c) after contact

References

(1) Sawicka-Chudy, P.; Sibiński, M.; Rybak-Wilusz, E.; Cholewa, M.; Wisz, G.; Yavorskyi, R. Review of the development of copper oxides with titanium dioxide thin-film solar cells. *AIP Advances* **2020**, *10* (1). DOI: 10.1063/1.5125433 (acccessed 1/29/2024).

(2) Koffyberg, F. P.; Benko, F. A. A photoelectrochemical determination of the position of the conduction and valence band edges of p-type CuO. *Journal of Applied Physics* **1982**, *53* (2), 1173-1177. DOI: 10.1063/1.330567 (acccessed 1/29/2024).

(3) Kashiwaya, S.; Morasch, J.; Streibel, V.; Toupance, T.; Jaegermann, W.; Klein, A. The Work Function of TiO2. *Surfaces* **2018**, *1* (1), 73-89.

(4) Setvin, M.; Hulva, J.; Parkinson, G. S.; Schmid, M.; Diebold, U. Electron transfer between anatase TiO₂ and an O₂ molecule directly observed by atomic force microscopy. *Proceedings of the National Academy of Sciences* **2017**, *114* (13), E2556-E2562. DOI: doi:10.1073/pnas.1618723114.

(5) Conesa, J. C. Modeling with Hybrid Density Functional Theory the Electronic Band Alignment at the Zinc Oxide–Anatase Interface. *The Journal of Physical Chemistry C* **2012**, *116* (35), 18884-18890. DOI: 10.1021/jp306160c.
